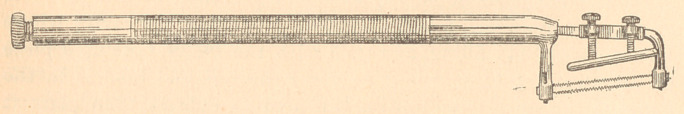# Saw-Frame and Gum-Guard

**Published:** 1892-02

**Authors:** W. H. Rollins


### SAW-FRAME AND GUM-GUARD.

BY DR. W. H. ROLLINS.

THE little saws we use about the teeth are perforated at the ends for the passage of small pins which hold the saw. On this account these saws are very weak and break constantly. The object of the frame to be described is to overcome this difficulty and to furnish the means of using a saw when broken. By means of the milled head the distance between the jaws of the frame can be varied to suit the length of the saw. Each of the jaws is perforated for a slot through which the saw passes and by a hole for the insertion of the little pins which hold it. To adjust a saw, remove the pins, put it in place, arrange the jaws to the right length, then press in the pins, which, bearing against the saw laterally, press it so firmly that it cannot get out. It is necessary to remove the temper from the saw at each end for an eighth of an inch, so the pins can dent it laterally in order to hold it firmly. I recommend the use of short saws, and find that three-quarters of an inch is the longest distance which the jaws should be apart, so I have directed Messrs. Codman & Shurtleff to make the frames to open this amount. The milled heads on the gum-guards are to turn the guard up and down, to adjust the frame so the saw cannot plunge into the gum.

## Figures and Tables

**Figure f1:**